# Formation of the Food Vacuole in *Plasmodium falciparum*: A Potential Role for the 19 kDa Fragment of Merozoite Surface Protein 1 (MSP1_19_)

**DOI:** 10.1371/journal.pone.0003085

**Published:** 2008-08-29

**Authors:** Anton R. Dluzewski, Irene T. Ling, John M. Hopkins, Munira Grainger, Gabriele Margos, Graham H. Mitchell, Anthony A. Holder, Lawrence H. Bannister

**Affiliations:** 1 Department of Immunobiology, Guy's, King's and St. Thomas' School of Medicine, Guy's Hospital, London, United Kingdom; 2 Centre for Ultrastructural Imaging, Guy's, King's and St. Thomas' School of Biomedical and Life Sciences, Guy's Hospital, London, United Kingdom; 3 Division of Parasitology, NIMR, Mill Hill, London, United Kingdom; London School of Hygiene & Tropical Medicine, United Kingdom

## Abstract

*Plasmodium falciparum* Merozoite Surface Protein 1 (MSP1) is synthesized during schizogony as a 195-kDa precursor that is processed into four fragments on the parasite surface. Following a second proteolytic cleavage during merozoite invasion of the red blood cell, most of the protein is shed from the surface except for the C-terminal 19-kDa fragment (MSP1_19_), which is still attached to the merozoite via its GPI-anchor. We have examined the fate of MSP1_19_ during the parasite's subsequent intracellular development using immunochemical analysis of metabolically labeled MSP1_19_, fluorescence imaging, and immuno-electronmicroscopy. Our data show that MSP1_19_ remains intact and persists to the end of the intracellular cycle. This protein is the first marker for the biogenesis of the food vacuole; it is rapidly endocytosed into small vacuoles in the ring stage, which coalesce to form the single food vacuole containing hemozoin, and persists into the discarded residual body. The food vacuole is marked by the presence of both MSP1_19_ and the chloroquine resistance transporter (CRT) as components of the vacuolar membrane. Newly synthesized MSP1 is excluded from the vacuole. This behavior indicates that MSP1_19_ does not simply follow a classical lysosome-like clearance pathway, instead, it may play a significant role in the biogenesis and function of the food vacuole throughout the intra-erythrocytic phase.

## Introduction

Most studies on merozoite surface protein 1 (MSP1) have focused on its role in erythrocyte invasion and therefore its potential as a vaccine candidate, based on the ability of MSP1-specific antibodies to inhibit invasion. However, it is known that a fragment of MSP1 (MSP1_19_) is carried into the erythrocyte during invasion and may persist for some time [1], [2]. The investigation reported here is focused on the possibility that MSP1_19_ may play a role in the biology of the intracellular stages.

MSP1 is synthesized by intracellular *Plasmodium falciparum* schizonts as a ∼200-kDa glycosylphosphatidyl inositol (GPI)-linked precursor, which is directed to the parasite's surface (a process requiring specific trafficking sequences [3]). Upon release of free merozoites the precursor is cleaved to four fragments of 83, 30, 38 and 42 kDa that remain associated and form a complex together with fragments of two other proteins, MSP6 and MSP7, on the merozoite surface. This location of MSP1 on the surface has been established biochemically, by immunofluorescence assay (IFA) and by immuno-electronmicroscopy (IEM) techniques. The localization of MSP1 to the merozoite surface in *Plasmodium yoelii* was one of the earliest successful applications of IEM to malaria parasites [4], and IEM was also used later to demonstrate the presence of this molecule on the surface of *P. falciparum* merozoites [5]. At the time of red blood cell (RBC) invasion a second proteolytic cleavage of the 42-kDa polypeptide, by the enzyme SUB2 [6], releases the protein complex from the parasite surface except for a 19-kDa C-terminal GPI-linked fragment (MSP1_19_). The latter comprises two epidermal growth factor (EGF)-like domains and is carried into the interior of the infected-RBC on the merozoite surface [7], MSP1_19_ has been detected on the surface of the early ring-stage parasite by both IFA [1], [2], and IEM [1]. Furthermore, antibodies specific to MSP1_19_ that are present in the culture medium at the time of invasion, can be internalized when bound to MSP1_19_ on the parasite surface [8]. However, the fate of MSP1_19_ after invasion has not been studied in any detail.

Available evidence indicates that invasion-related merozoite surface molecules are proteolytically cleaved at or immediately after invasion [9]–[14]. The fate of any resulting internalized fragments is poorly understood. Recently Drew et al [15] reported the detection of MSP1_19_ by IFA in the food vacuole of late rings/trophozoites, suggesting that this organelle is able to receive molecules endocytosed from the parasite surface. Although there is no clear morphological evidence for the existence of a classical eukaryotic endosome-lysosome system in *Plasmodium,* the food vacuole may act as a lysosome-like compartment as it contains proteases (see [16], [17]) able to degrade hemoglobin ingested from the RBC within an acidic environment [18].

The food vacuole is a highly specialized organelle, formed by endocytosis from the parasite surface via a cytoskeletal ring, the cytostome. Through this, RBC cytosol together with the attendant membranes of the parasitophorous vacuole and parasite surface (its plasma membrane), are internalized to form one or more food vacuoles [19]–[24]. These receive degradative enzymes from the parasite's secretory pathway [25] to break down the engulfed hemoglobin and release the iron-containing haem component (hematin), which is dimerized to β-haematin and crystallizes as the chemically inert malaria pigment, hemozoin [26]–[28].

Early after invasion, numerous small food vacuoles form within the ring stage parasite [29] replaced later by a single large food vacuole, which eventually becomes filled with hemozoin crystals. A well-studied marker for the food vacuole membrane, the chloroquine resistance transporter CRT [30], [31] (see [32] for review) is predicted to be a transporter protein (a member of the drug/metabolite superfamily [33], [34]), spanning the food vacuole membrane [31]. Another molecule localized to the food vacuole is the so-called merozoite surface protein 8 (MSP8) [15], [35], [36] which, despite its name, is synthesized in ring stages. It is transported initially to the parasite's plasma membrane and thence, as a processed form, to the single food vacuole of late rings/early trophozoites [15].

In the present study we have explored in detail the post-invasion fate of MSP1_19_ using a combination of metabolic labeling, IFA and IEM techniques applied to highly synchronized parasite cultures, together with antibodies directed against well-defined regions of MSP1. We find that after endocytosis into small food vacuoles in the ring stage, MSP1_19_ persists through the cycle as a coherent component of the food vacuole wall without further processing or addition of newly synthesised MSP1. This behavior indicates that the molecule does not simply follow a classical lysosome-like clearance pathway, but may play a significant role in the biogenesis and function of the food vacuole throughout the intra-erythrocytic phase.

## Materials and Methods

### Parasites

*P. falciparum* lines 3D7 [37], C10 [38] and IT04 [39] were maintained *in vitro* as described [40] using either 10% human serum or 0.5% (w/v) AlbuMAX® I in RPMI 1640 medium. Parasites were synchronized using combinations of a magnet [41], plasmagel flotation [42] and/or Percoll gradient centrifugation to purify late-stage parasites [43], and sorbitol synchronization of ring-stage parasites as described previously [44].

### Antibodies

A number of antibodies against different regions of MSP1 were used: monoclonal antibody (mAb) 1E1 [8] specific for MSP1_19_; rabbit polyclonal antibodies raised against either affinity purified MSP1 (Wellcome type) [45] or recombinant GST-MSP1_19_ [46]; and a pool of rabbit polyclonal antibodies raised against recombinant protein epitopes spanning parts of MSP1 that are N-terminal of MSP1_19_ (i.e. not reacting with MSP1_19_) [47]. These latter recombinant proteins correspond to the following positions in the protein sequence (Acc. # X02919): pME3, residues 1046–1448; pME17, 1046–1204; pME18, 1232–1372; pME19, 1221–1250; and pME22 1046–1498. The first residue of MSP1_19_ is position 1526. Rabbit antibodies raised against peptides of CRT [31] were obtained from MR4 (courtesy of Drs. R. Cooper and T.E. Wellems) and as a kind gift from Prof. Leann Tilley (La Trobe University, Bundoora). For controls, normal rabbit and mouse antisera were used for western blots, IFA and IEM, plus a wide range of antibodies raised against other *P. falciparum* antigens, for IEM.

### Radiolabeling and immunoprecipitation

Mature stage parasites (3D7) were metabolically labeled with ProMix (^35^S Met/Cys; GE Healthcare, Little Chalfont, UK) essentially as described previously [45]. Synchronized parasites were labeled for 1.5 hours at 45 hours post invasion. After removal of the label, a portion of the parasites was harvested and stored at −70°C. The remainder was allowed to re-invade fresh erythrocytes for 2 hours and then any residual schizonts were removed. The parasites were divided into 8 equal portions. One was harvested immediately (time 0) and the remaining parasites returned to culture for harvesting at 6-hour intervals until 42 hours post-invasion. Giemsa-stained smears were made at each time-point as well as thin smears for IFA as described below.

For immunoprecipitation analysis, parasite extracts were made using buffer containing Nonidet P40 as described previously [45], [48]. Polyclonal anti-MSP1 (affinity purified) serum was used to precipitate proteins, which were then solubilized under reducing conditions and analysed by SDS-PAGE on 5–12.5% gradient gels. The gels were stained with Coomassie blue, treated with Amplify (GE Healthcare) for 30 minutes, dried at −70°C and exposed to X-ray film (Kodak, Rochester, NY) at −70°C for a suitable period.

### Fluorescence imaging

Thin blood films were made on microscope slides at intervals from time 0 (when newly invaded erythrocytes had been synchronized and returned to culture), air-dried, frozen without fixation and stored desiccated at −20°C until required. The time-course slides were assayed as follows: samples were thawed and dried quickly, fixed in fresh 1% formaldehyde in PBS for 30 minutes, rinsed in phosphate buffered saline (PBS), permeabilized with 0.1% Triton X-100/PBS for 10 minutes, rinsed in PBS and blocked overnight at 4°C in 3% BSA/PBS. Cells were probed consecutively with primary antibodies in 3% BSA/PBS, and with Alexa Fluor 488 (green) or Alexa Fluor 594 (red)-conjugated affinity purified goat anti-mouse IgG or anti-rabbit IgG secondary antibody (Molecular Probes) as appropriate at 37°C for 30 minutes each, followed by three washes in PBS. Dual labeling experiments were performed on thin films and probed successively with different primary antibodies derived from different species (mouse and rabbit). This was followed by staining with appropriate secondary antibody (anti-IgG) conjugated to Alexa Fluor 488 or Alexa Fluor 594. Control incubations were carried out in the absence of primary or secondary antibodies in addition to a range of antibody controls described above. Under no condition was a false fluorescence signal from the hemozoin observed. Finally, the films were stained with 0.5 µg ml^−1^ 4′, 6-diamidino-2-phenylindole (DAPI) in PBS to identify the nuclei. The films were subsequently mounted in glycerol/PBS solution containing anti-quenching agent (Citifluor Ltd., U.K.) and viewed under oil immersion. The parasites were viewed using a Zeiss Axioplan2 microscope equipped with a Plan-APOCHROMAT 100×/oil immersion lens and appropriate filters. Images were analysed and processed using Adobe Photoshop (Adobe Systems Inc., San José, CA) and Microsoft PowerPoint software.

To pre-label MSP1_19_ with antibody (i.e. prior to invasion) synchronous mature parasites were cultured and allowed to invade in the presence of mAb 1E1: mature schizonts were purified using a combination of gel flotation and Percoll purification. Fresh erythrocytes were added to give a parasitemia of 5% (at 17% hematocrit) and samples were split into two fractions; one-half was placed in normal medium; the other in medium containing 500 µg ml^−1^ mAb 1E1. Following further culture with gentle shaking for 1 hour, remaining schizonts were removed using a Percoll cushion followed by sorbitol treatment of the pellet. A sample was taken (t = 0) and smeared for Giemsa staining and for IFA, then the remaining parasites were cultured and smears were prepared every 3 hours over a 48-hour time period. The overall window for invasion was 2 hours.

### Electron microscopy

Schizonts (*P. falciparum* clones 3D7 and C10, and line IT04) were prepared for IEM as described in [45]. Briefly, schizonts were enriched on Percoll, then either fixed immediately or incubated in the presence of fresh erythrocytes for up to 2 hours to allow the study of early ring stages. Cells were fixed in 0.1% v/v glutaraldehyde and 2% w/v paraformaldehyde made up in RPMI 1640 medium for 20 minutes at 4°C. Samples were dehydrated by the progressive low temperature method, embedded in LR White resin (Agar Scientific UK), and polymerized at room temperature by UV light. For immuno-staining, MSP1-specific rabbit polyclonal antibodies were used, followed by protein-A conjugated to 10 nm gold, a kind gift from Dr. Pauline Bennett, King's College London. For controls, parallel sets of sections were incubated with pre-immune sera for the primary antibody step, or with antibodies raised against a wide range of merozoite rhoptry, micronemal and dense granule proteins. Immuno-stained sections were contrasted with 2% aqueous uranyl acetate. For morphology, cells were fixed in 2.5% v/v glutaraldehyde in 0.08 M sodium cacodylate buffer (pH 7.2), and processed as described in [49]. Epoxy resin sections were stained with uranyl acetate and lead citrate then examined in a Hitachi 7600 electron microscope. Serial sectioning was also carried out on ring-stage material, using a Reichert Ultracut E ultramicrotome. Section ribbons were collected on pioloform-support films covering slotted grids, then stained and viewed as above.

## Results

### Radiolabeling and immunoprecipitation

**Figure 1:**
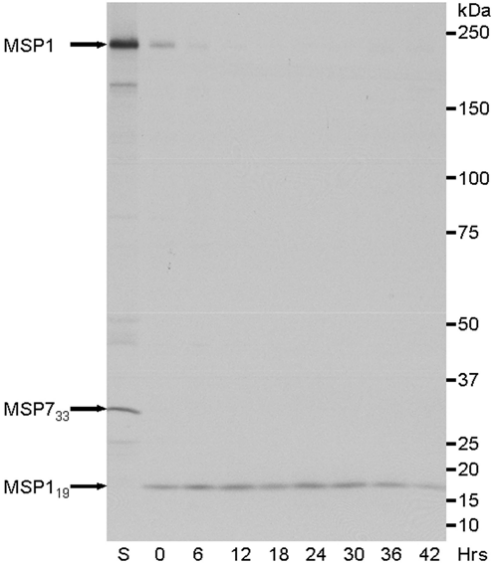
Radiolabeling of MSP1_19_, the final processed fragment of MSP1, showing that it remains attached to the parasite after merozoite invasion of erythrocytes, and that it persists within the parasite throughout its life cycle. *P. falciparum* schizonts were radiolabeled with ^35^S Met/Cys at 45 hours post-invasion for 1.5 hour and a proportion of these parasites were harvested (S, schizonts). The remaining parasites were allowed to undergo invasion, synchronized (2-hour window) and cultured for the next intraerythrocytic cycle. Parasites were harvested every 6 hours from 0 to 42 hours. Rabbit anti-MSP1-specific antibodies were used to immunoprecipitate labeled protein from NP40-extracts of these parasites. The resulting precipitates were analyzed by SDS-PAGE using a 5–12.5% gradient gel under reducing conditions and visualized by fluorography using X-ray film. The polyclonal anti-MSP1 serum recognized the full-length precursor as indicated by the top arrow in the labeled schizont extract, (S), and the 19-kDa fragment in newly formed ring stages (0 hours), indicated by the bottom arrow. The processed fragment of MSP7, MSP7_33_ is also detected as part of the MSP1 complex in schizonts (middle arrow). The remaining lanes represent parasites harvested every 6 hours from 6 to 42 hours post-invasion. The intensity and the mobility of the MSP1_19_ band remained constant until 42 hours when there was some decrease in intensity; this reduction in intensity could be due to rupture of some mature forms releasing MSP1_19_ into the culture medium. The mobility of protein size markers is indicated at the right of the panel according to their size (kDa).

The persistence of MSP1_19_ during intraerythrocytic development was examined by biosynthetically labeling MSP1 during the schizont stage and then establishing the presence of the protein in the next intracellular cycle by immunoprecipitation. From extracts of labeled schizonts, MSP1-specific antibodies precipitated the ∼200-kDa full-length precursor but not MSP1_19_ (Figure 1 lane S, indicated by the top arrow). In addition, the 33-kDa intermediate processing fragment of MSP7 that is tightly associated with MSP1 [50], was also present in this immunoprecipitate (indicated by the middle arrow, Figure 1). In contrast, the same antibodies precipitated only MSP1_19_ from ring stages and throughout further intracellular development, at least up until 42 hours post invasion (Figure 1, lanes 0 to 42 hours, indicated by lower arrow). Both the apparent molecular mass and intensity of the MSP1_19_ band remained constant during maturation of the parasite until the end of the cycle, when there was some reduction in signal strength. Importantly, the integrity of MSP1_19_ throughout the erythrocytic cycle indicates that this protein was not further modified or degraded during intracellular development. The reduction in signal at the end of this cycle is most likely attributable to the beginning of schizont rupture.

### Fluorescence imaging

Indirect immunofluorescence was used to study the location and persistence of MSP1_19_ during the parasite's intracellular development. In particular the location of MSP1_19_ was compared with that of CRT, a protein that is a marker for the food vacuole.

In the first series of experiments MSP1 on the merozoite surface was tagged by allowing parasites to invade erythrocytes in the presence of the mAb 1E1, which binds to MSP1_19_, and is known to be carried into the RBC with the merozoite during invasion [8]. In the presence of mAb 1E1 parasites invaded red cells and developed normally compared to untreated controls (data not shown). At various times after invasion parasites were fixed and the bound antibody detected by IFA using fluorescently labeled affinity-purified anti-mouse IgG. The fixed parasites were also incubated with either a MSP1_19_-specific rabbit polyclonal antibody or an antibody specific for the CRT protein, and the binding of these antibodies was detected with appropriately labeled second antibodies.

**Figure 2:**
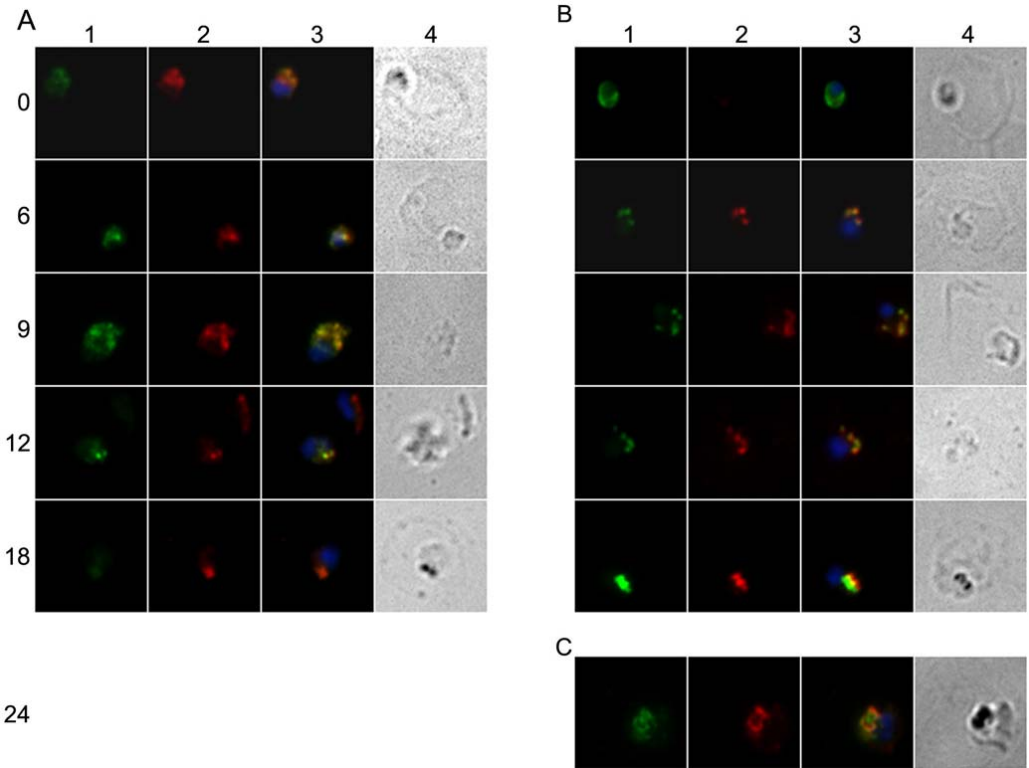
Immunofluorescence microscopy showing the localization of MSP1_19_ and CRT in parasites that had invaded in the presence of mAb 1E1; showing that MSP1_19_ moves to the food vacuole. Schizonts were allowed to release merozoites that invaded fresh erythrocytes in the presence of mAb 1E1, then ring-stage parasites were cultured and samples harvested every 3 hours over the asexual cycle. The mAb 1E1 antibody associates with MSP1_19_ on the surface of the merozoite, and is detected within the ring-stage parasite; it was used to follow MSP1_19_ in smears counterstained with rabbit anti-MSP1_19_ (panel A) and rabbit anti-CRT (panel B), which is a marker of the food vacuole. Each row of four panels show an identical field from 1% formaldehyde-fixed thin smears of *P. falciparum* ring-stage parasites; columns A1 and B1 show mAb 1E1 associated with the parasite detected with Alexa Fluor 488 conjugated anti-mouse IgG (green); columns A2 rabbit anti-MSP1_19_ and B2 rabbit anti-CRT, detected with Alexa Fluor 594 conjugated anti-rabbit IgG (red); columns A3 and B3 are composites of columns 1 and 2 with DAPI staining of the nucleus (blue) and any green and red fluorescence overlap is displayed in yellow. In columns A4 and B4, the parasitised erythrocyte is visualized by light microscopy, showing the location of the parasite within the infected erythrocyte. Only five time points are shown: 0, 6, 9, 12 and 18 hours post-invasion. The two anti-MSP1_19_ antibodies, in panels A1 and A2, are seen to co-localize in panel A3, showing that 1E1 is still associated with MSP1_19_ in the young parasite. CRT is clearly detectable from 6 to 9 hours onwards post-invasion (panel B2) and appears to be closely associated with MSP1_19_ (panel B3). Pigment granules are clearly visible by light microscopy from about 18 hours (panels A4 and B4). Similar results were obtained in a separate experiment in which mAb 1E1 was used to detect MSP1_19_ on formaldehyde fixed parasites at the same time points (data not shown) and at 24 hours (panel C). Panels C1–C4 show 1E1, (C1, green), rabbit anti-CRT (C2, red), composites of these antibodies with DAPI staining merged (C3) and light visualization (C4), as described above. CRT was clearly detectable (C2) and largely co-localized with MSP1_19_ (C3). Both MSP1_19_ and CRT were associated with the pigment detected by light microscopy in panel C4 and no longer around the surface of the parasite.

In newly invaded parasites, MSP1_19_ was clearly visible as a bright green peripheral staining (Figure 2, time 0). As the young parasite developed within the erythrocyte the pattern of MSP1_19_ changed quite quickly, becoming punctate, but still remaining at or near the periphery of the parasite (Figure 2, 6 to 9 hours). However, from around 18 hours, the staining became increasingly concentrated in a small number (two to four) of prominent foci. A comparison of the location of MSP1_19_ with that of hemozoin, detected under bright field illumination and visible from the late ring stage (18 hours) onwards indicates that the protein is associated with food vacuoles, and that the small food vacuoles had increased in size but decreased in number, suggesting their fusion. The subcellular location of mAb 1E1 bound to MSP1_19_ was identical to that of an MSP1_19_-specific polyclonal antibody that was applied to the parasites post-fixation (Figure 2 panel A). This co-localization persisted throughout ring-stage development, showing that mAb 1E1 remained associated with MSP1_19_ within the parasite. However during this development the intensity of the 1E1 label progressively diminished, perhaps indicating a progressive degradation of the antibody as the parasite matured.

To determine if MSP1_19_ is present in the food vacuole we examined its colocalization with CRT by IFA. We found that CRT was first detectable in rings at around 6 hours post invasion in the form of diffuse staining at the periphery of the parasite, with some punctate staining (Figure 2, panel B). From 12 hours post-invasion CRT labeling was concentrated in several small bright foci, still close to the periphery of the parasite, suggesting a cluster of small food vacuoles. From 18 hours post invasion hemozoin pigment was also visible within these vacuoles. In these double-labeled samples, the CRT-specific fluorescence coincided closely with MSP1_19_-specific fluorescence. The appearance and location of CRT-specific label was unaffected by the presence of the internalized mAb 1E1. By 24 hours hemozoin crystals were clearly evident in a single food vacuole and were surrounded by both MSP1_19_ and CRT fluorescence (Figure 2, panel C). However, close inspection showed that while there was considerable co-localization of MSP1_19_ and CRT at this stage, coincidence was not complete.

**Figure 3:**
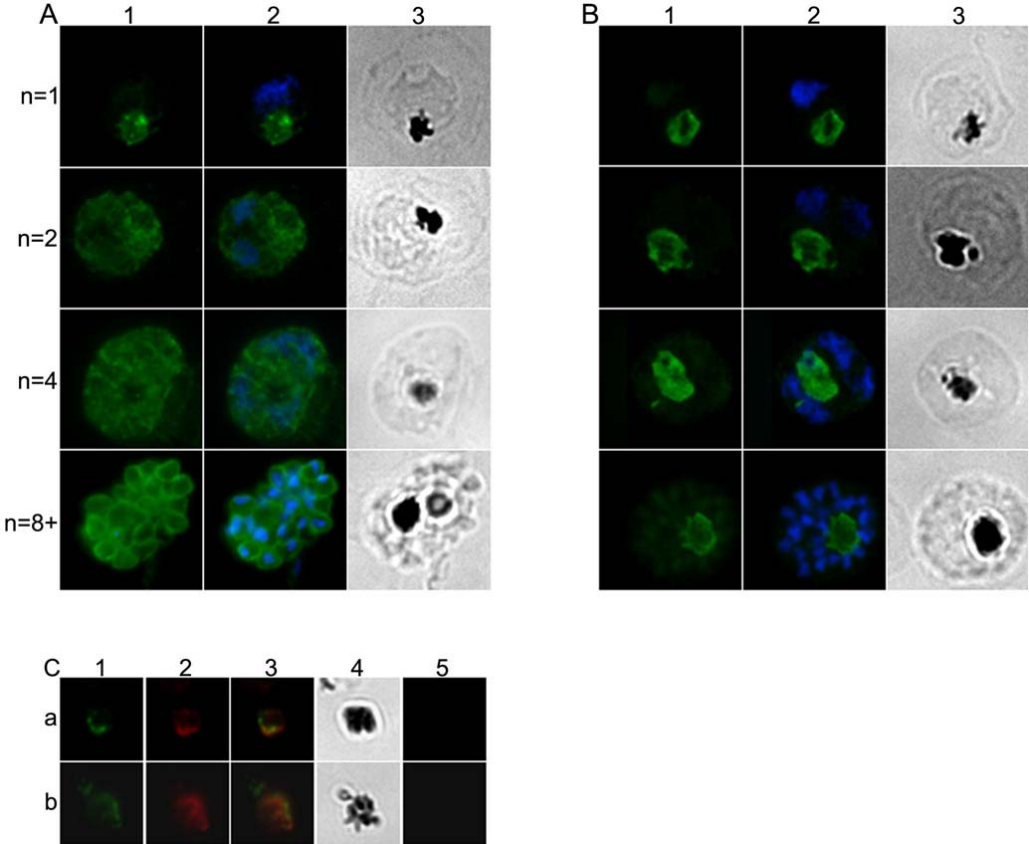
IFA location of MSP1_19_, CRT and new full length MSP1 during schizogony. Mature parasites at different stages of nuclear division were compared. Each row in panels A and B shows an identical field from 1% formaldehyde-fixed thin smears of late-stages parasites at the 1-, 2-, 4-, and 8-nuclei stage. Panels A1 and B1 show anti-MSP1_19_(rabbit) and rabbit anti-CRT(rabbit) antibody labeling respectively (the secondary antibody was Alexa Fluor 488-conjugated anti-rabbit IgG antibody (green). Panels A2 and B2 show the same fields merged with the corresponding DAPI stained nucleus images (blue). Panels A3 and B3 show the same fields visualized by light microscopy - note the pigment is clearly visible. Rabbit-anti-MSP1_19_ antibody only detects MSP1_19_ associated with the food vacuole until the two-nuclei stage. Once new full length MSP1 is synthesized and transported to the parasite's plasma membrane the presence of MSP1_19_ from the previous cycle is now obscured. In contrast, rabbit-anti-CRT antiobody is clearly detectable throughout parasite maturation and is associated with the food vacuole. Panel C, rows (a) and (b), shows that MSP1 and CRT are associated with the residual body that is released upon schizont rupture. Thin smears made at the time of schizont rupture were probed consecutively with mAb 1E1 and rabbit anti-CRT antibodies. These antibodies were detected using Alexa Fluor 488-conjugated anti-mouse IgG, and Alexa Fluor 594-conjugated anti-rabbit IgG, respectively (columns 1 and 2). Column 4 shows the bright field microscopy images of the same field and clearly indicate the presence of malarial pigment. Column 5 shows the absence of nuclear material in the residual bodies, as confirmed by the lack of DAPI staining. Column 3 shows the merged images from columns 1, 2 and 5 - both CRT and MSP1_19_, or MSP1, which cannot be distinguished by the antibody, are associated with the released pigment in the residual body.

In a second series of experiments the locations of MSP1_19_ and CRT were examined in later stages (Figure 3). MSP1_19_ and CRT were clearly visible surrounding the hemozoin in a late trophozoite (with a single nucleus) (Figure 3, panels A and B top row). As the parasite matured and the number of nuclei increased, the pattern of CRT staining of the food vacuole remained constant (Figure 3, panel B). At the same time, the increasing expression of newly synthesized MSP1 at the schizont surface [51] reacting with the anti-MSP1_19_ antibodies progressively obscured the MSP1_19_-specific signal associated with the food vacuole (Figure 3, panel A). At the end of schizogony when the residual body containing the hemozoin pigment is released together with the merozoites, the residual body reacted with both MSP1- and CRT-specific antibodies (Figure 3, panel C, (a) and (b)), however MSP1_19_ could not be distinguished from newly synthesized MSP1 by these fluorescence techniques.

In agreement with the biosynthetic labeling data, MSP1_19_ was detectable in fixed parasites in two independent experiments with rabbit polyclonal anti-MSP1_19_ and mAb 1E1 antibodies at least until 36 and 30 hours post-invasion, respectively, after which time the presence of newly synthesized MSP1 on the parasite surface masked the labeling of internalized MSP1_19_ (Figure 3).

### Immuno-electronmicroscopy

The ultrastructure of the *P. falciparum* food vacuoles at different blood cycle stages has been previously reported (see Introduction), but to help the description of our IEM findings, we will briefly describe their morphology in rings and then in later stages.

#### The endocytic vacuoles in rings (Figures 4A–G and 5A–F)

**Figure 4:**
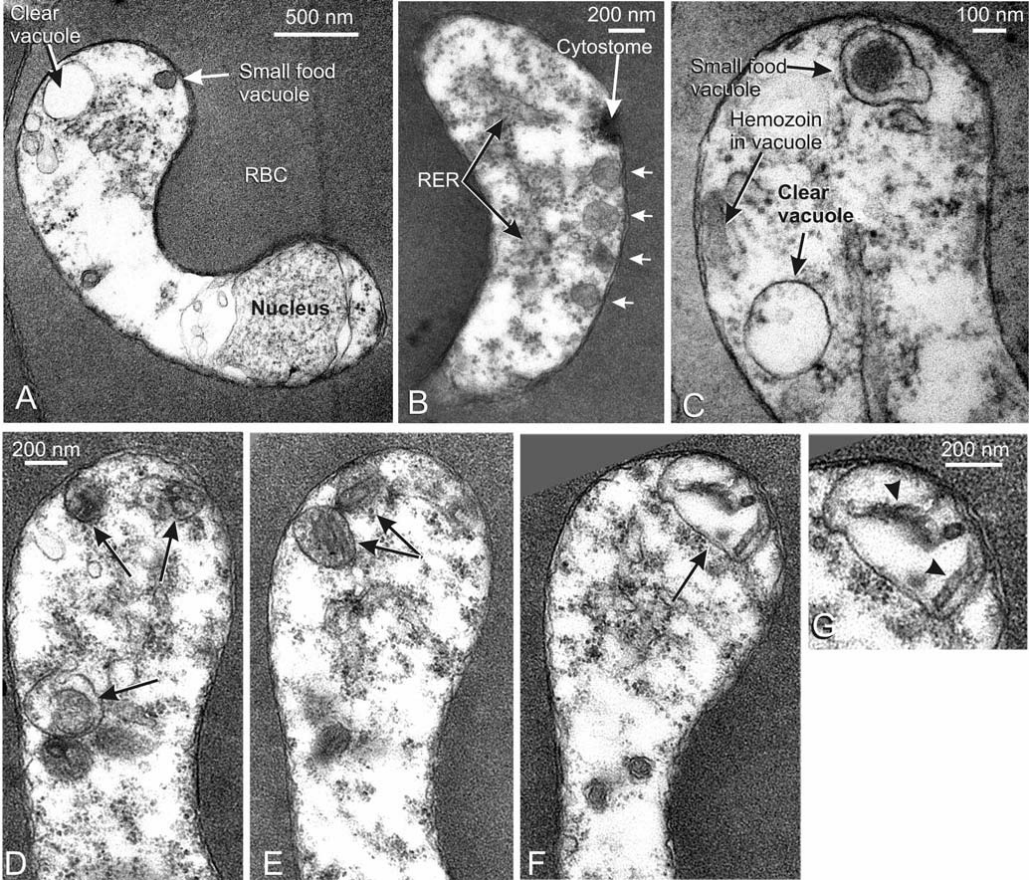
Ultrastructural morphology of endocytic vacuoles in ring stages. Panels A to G show sections through established ring stages prepared for EM morphology, A and C show the two types of vacuole present in the cytoplasm, one not containing hemoglobin (clear vacuoles) and the other with hemoglobin (small food vacuoles). An obliquely sectioned cytostome and a group of small dense food vacuoles are visible in B. In C a double membrane dense food vacuole is also shown, which is typical of early endocytosis before the inner membrane breaks down (see text). In panels D–F three sections from a serial section sequence are shown, depicting stages in hemozoin formation in a mid-stage ring. In panel D a vacuole in the early stages of hemoglobin degradation is visible (arrow, bottom left) as well as smaller vacuoles containing dense masses indicating hemozoin formation, In panel F a larger vacuole containing a number of small hemozoin crystals is shown (enlarged in panel G). *Abbreviations*: RER–rough endoplasmic reticulum; RBC–red blood cell.

**Figure 5:**
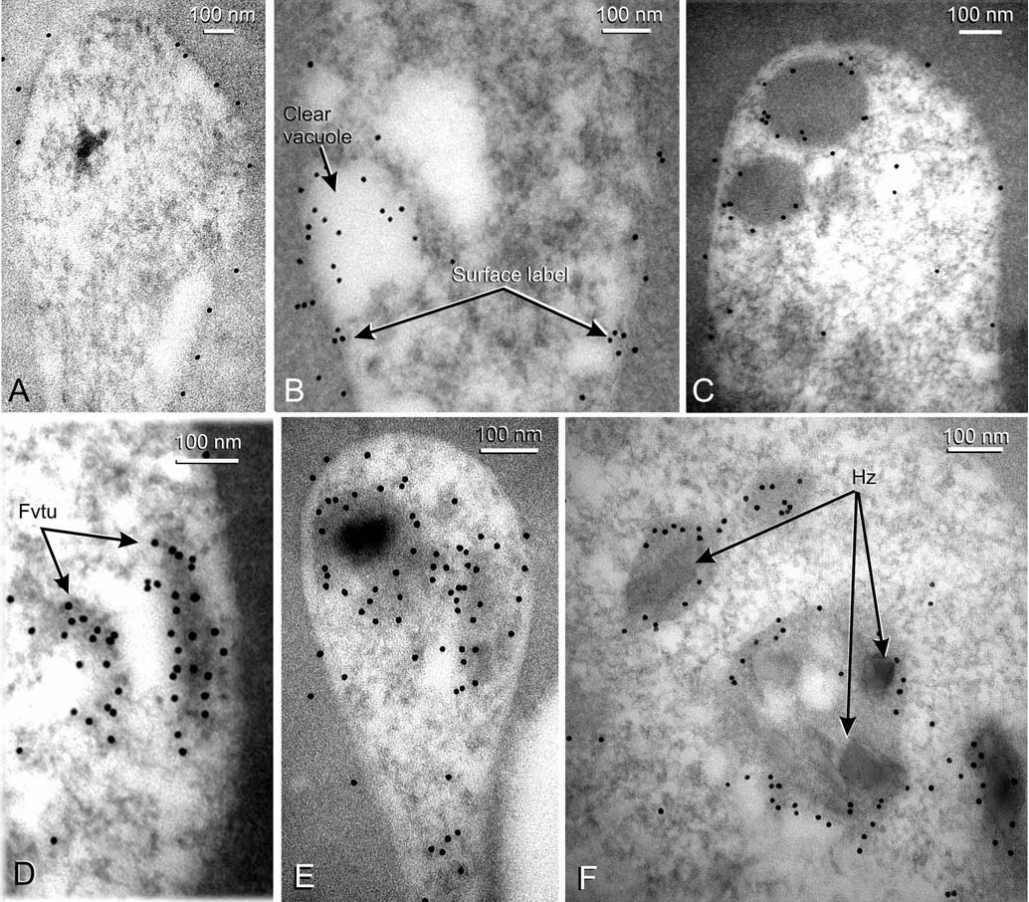
The distribution of MSP1_19_ in ring-stage parasites detected by IEM. Panels D and E show MSP1_19_-specific labeling of the surface of early rings, and in panel E, a clear endocytic vacuole is also labeled. Panels F–H show that MSP1_19_ has been endocytosed into the small dense food vacuoles, and is located mainly at the inner vacuolar membrane surface; in Panel G the food vacuoles have a tubular appearance. In Panel I, a late ring is labeled, showing the incorporation of MSP1_19_ into larger vacuoles containing small hemozoin crystals (Hz), with the label again associated with the vacuole membrane. *Abbreviations*: Fvtu- tubular food vacuoles; Hz- hemozoin.

In early to mid-stage rings, EM showed endocytosis of erythrocyte cytoplasm into small dense vacuoles with diameters of 150–300 nm (Figure 4A, B). Some vacuoles were a little larger (350 nm) and surrounded by two membranes (Figure 4C, D), indicating that they had just been endocytosed, with the inner membrane being derived from the parasitophorous vacuole membrane with enclosing red cell cytoplasm, and the outer membrane derived from the parasite plasma membrane (see [22]). Only a single enclosing membrane was present around most dense vacuoles, denoting lysis of the inner membrane. Some vacuoles contained one or more small electron-dense masses showing the formation of crystals of hemozoin instead of the dense granular contents (Figure 4C–G), indicating that hemoglobin degradation had begun soon after invasion. In addition, some early rings contained one or more clear single membrane vacuoles (Figure 4A, C), but these did not persist in later rings. In mid- and late-stage rings, larger vacuoles containing several hemozoin crystals (Figures 4F, G, and 5F) were present, suggesting that progressive vacuolar fusion was occuring. In very late ring forms, as in trophozoites and schizonts, all hemozoin was contained in a single larger vacuole. Although single, random sections through rings did not always contain vacuoles with hemozoin, all serially sectioned sequences (see e.g. Figure 4D–F) (seven in total) and numerous partial sequences showed their presence. The small vacuoles were typically clustered together locally just beneath the plasma membrane (e.g. Figure 4B), so that random sections could easily miss passing through such an area. The characteristic cytostomal apparatus consisting of an electron-dense cytoskeletal double ring attached to the parasite's plasma membrane (Figure 4B), was present in all stages from early rings onwards.

IEM with the polyclonal anti-MSP1_19_ antibody in early ring stages showed labeling present over the whole surface of the parasite's plasma membrane (Figure 5A, B) and occasionally within clear vacuoles within the parasite (Figure 5B). However, in most rings, labeling was confined to the small dense food vacuoles and tubular food vacuoles situated near the parasite's periphery (Figure 5C–E), and, in more mature rings, to the larger more central vacuoles (Figure 5F), none of which were unlabeled. In all ring stages, labeling was located along the inner surface of the food vacuole membrane. When antibody raised against the N-terminal end of MSP1 (i.e. excluding the MSP1_19_ region) was used, no labeling was found on any ring structure (data not shown).

#### Endocytosis in later-stage parasites (Figure 6A–J)

**Figure 6:**
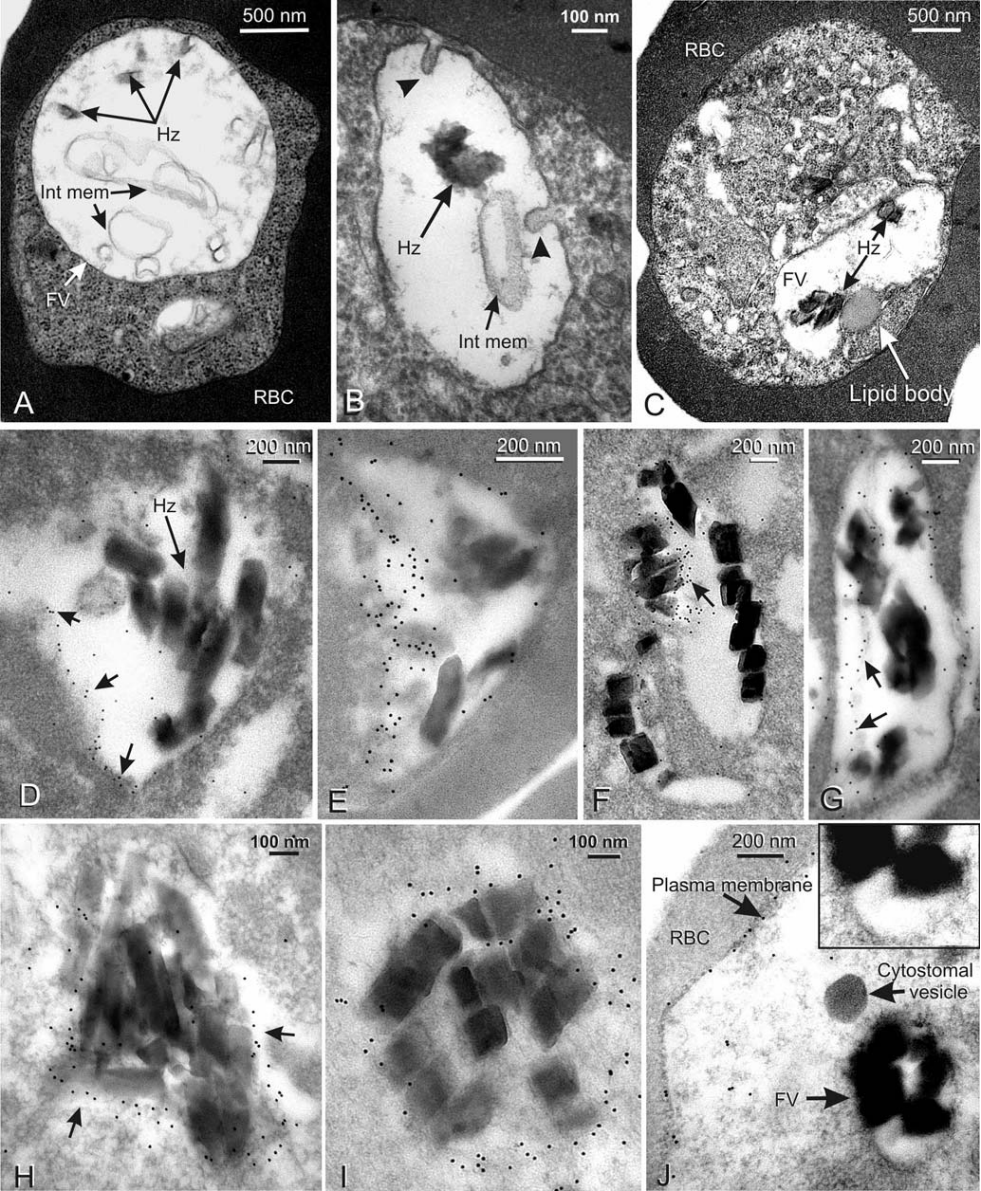
The distribution of MSP1 within late trophozoites and schizonts. Panels A–C show the appearance of the single food vacuole, prepared for EM morphology. Panel A shows a late trophozoite containing a typically dilated food vacuole, containing widely spaced hemozoin and several profiles of internal membranous structures. In panel B an early schizont stage vacuole contains some hemozoin crystals and internal membrane profiles, while the vacuole wall shows signs of inward folding (arrowheads). C shows another schizont where a lipid body has formed adjacent to the food vacuole. Panels D–G show specific immunogold labeling of early to mid-stage schizonts labeled with MSP1_19_-specific antibodies, in all cases detecting the protein at the vacuole wall. In panel D a limited area of labeling is present along one side of the food vacuole (small arrows), and in E a similar distribution is seen on a more folded vacuole wall. Panels F and G show labeling along obliquely sectioned folds of the wall (arrows), where the label also lies close to hemozoin crystals. Panels H and I show food vacuoles of late-stage schizonts containing closely packed large hemozoin crystals and are almost completely surrounded by MSP1_19_ labeled vacuole wall membrane. In Panel J (and the inset of a portion at higher magnification) an early-/mid-stage schizont has been immunostained with antibody reacting with N-terminal regions of MSP1 but not MSP1_19_. This antibody labels the newly synthesized (full-length) MSP1 on the parasite's plasma membrane but fails to label the food vacuole, indicating that the MSP1_19_-specific labeling, seen in panels D–I, is specific for MSP1_19_ carried in on the parasite surface at invasion rather than new MSP1 recently synthesized by the schizont. *Abbreviations*: FV–food vacuole; Hz–hemozoin; Int mem–internal membranous structures; RBC–red blood cell.

Morphological EM of trophozoites and schizonts, in which a single vacuole is present in each parasite, showed that food vacuole size varied with post-invasion age. In trophozoites and early schizonts the vacuole was rounded or elliptical in section and larger than in rings (up to 2.2 µm across, see e.g. Figure 6A), containing widely separated hemozoin crystals, and various irregularly shaped membranous sacs (referred to here as internal membranes, Figure 6A, B). In the mid-schizont period prior to merozoite budding the vacuole was somewhat smaller and the vacuole wall was often inwardly folded (Figure 6B), sometimes to create pockets partially enclosing hemozoin crystals. At this schizont stage, one or more lipid bodies associated with the external surface of the vacuole membrane were present (Figure 6C) (see [52]). In more mature, budding schizonts, the food vacuole was markedly smaller (0.8–1.2 µm in maximum diameter), its membrane closely fitting the cluster of larger tightly packed hemozoin crystals typical of the mature schizont (Figure 6I). In these parasites the lipid bodies were no longer visible.

IEM of trophozoites and early schizonts showed strong MSP1_19_-specific labeling, closely associated with part of the vacuole's lining membrane including its inward folds (Figure 6A–G) and often lying close to the hemozoin crystals (Figure 6F). In segmenting and later schizonts (Figure 6H, I) when the folds had mostly disappeared, labeling was still tightly related to the vacuole wall. It was noticeable that, except in the most mature schizonts, labeling was concentrated over a limited area of the vacuole membrane, thus some relatively large areas were devoid of immunostaining (Figure 6D–G). However, towards the end of the schizont period, label covered most or all of the inner surface of the vacuole membrane (Figure 6H, I).

We also carried out IEM with a mixture of polyclonal antibodies directed against the N-terminal portion of MSP1 that did not react with MSP1_19_. These labeled the plasma membrane of mid- to late-stage schizonts expressing newly synthesized MSP-1 on their surface, but failed to label the food vacuole or cytostomal vesicles en route to that organelle (Figure 6J). IEM controls using normal mouse or rabbit serum, or antibodies against irrelevant proteins, were all negative. We also attempted to detect CRT by IEM, but the labeling frequency was too low to provide useful data for this study.

## Discussion

**Figure 7:**
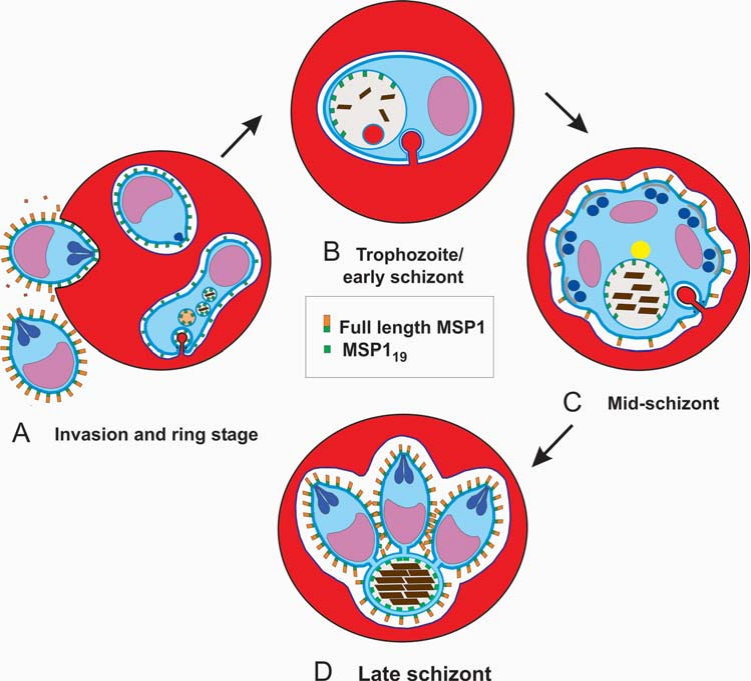
A diagram summarising the general fate of MSP1 and the C-terminal fragment MSP1_19_ through the asexual cycle. In panel A, MSP1 on the surface of the invading merozoite undergoes cleavage to release the N-terminal portion (orange) and leave the C-terminal MSP1_19_ (green) attached to the surface of the parasite: firstly as a newly internalized merozoite; and later as it transforms into a ring stage. MSP1_19_ is then endocytosed from the ring surface into small food vacuoles as the parasite begins to feed on and metabolise hemoglobin. In B, all the MSP1_19_ has been endocytosed and is associated with the membrane of the now single food vacuole, where it remains through the mid-schizont stage (C) and is then incorporated into the residual body at the end of schizogony (D).

**Figure 8:**
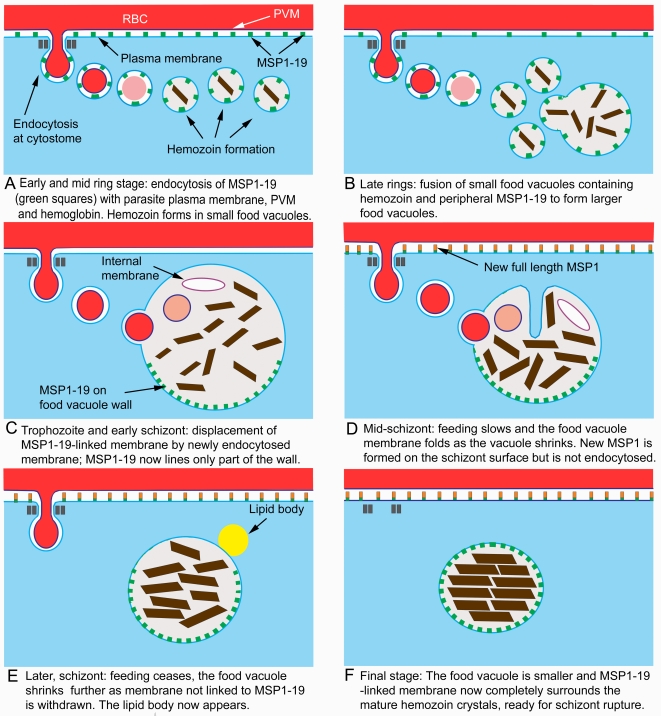
A series of diagrams summarising in greater detail the main conclusions relating to post-invasion MSP1_19_ endocytosis and its progression through the asexual cycle. In each panel a portion of the parasite is shown in blue, with the adjacent RBC in red, and with the parasitophorous vacuole space between the two. The fragments of MSP1 are colored as in Figure 7.

MSP1_19_ is formed by the cleavage of MSP1_42_ by SUB2 on the surface of the merozoite at the time of erythrocyte invasion [6]. We have followed the fate of MSP1_19_ in the subsequent intracellular development of the parasite and suggest that it plays an important but as yet undefined role in the food vacuole. Our conclusions are depicted diagrammatically in Figures 7 and 8.

The radiolabeling results show that after invasion MSP1_19_ does not undergo further processing or degradation within the intracellular parasite and is still intact at the point of merozoite release. The metabolic labeling results also show that new MSP1_19_ fragments are not formed by proteolytic cleavage from newly synthesized MSP1, so in schizonts all the MSP1_19_ must have entered from the previous parasite generation, during merozoite invasion. MSP1_19_ is known to be highly resistant to proteases, including those present in acidic lysosomal compartments [53], probably due to its compact, highly disulphide-bonded structure [54], [55]. In this context it is of interest that MSP8 which is synthesized in the ring stage and remains throughout the erythrocytic life cycle [15], has a similar double EGF-domain structure which may also protect it against proteolysis. Whether or not any other specific invasion-related merozoite proteins follow this route will be interesting to determine. The merozoite rhoptry proteins of the RhopH complex including RhopH1/Clag 9 [45] and RhopH2 [56] can be detected within the infected-RBC after invasion in association with the parasitophorous vacuole membrane, but there is no reported evidence of their endocytosis by the parasite.

Both IFA and IEM methods show that MSP1_19_ is cleared from the ring-stage parasite's surface by endocytosis into the vacuolar system where it remains until the schizont breaks down at the release of the next generation of merozoites. It is interesting however that in very early rings, some MSP1_19_ also enters a few clear vacuoles lacking hemoglobin or hemozoin, suggesting an early phase of endocytosis that does not involve RBC feeding (see also [29]). Nevertheless this appears to be a relatively minor, transient fraction restricted to the earliest rings, and since later labeling is only present in the dense small food vacuoles, the clear vacuoles must presumably transfer their contents to the dense ones. Therefore essentially, MSP1_19_ is endocytosed along with RBC cytosol into the multiple small dense vacuoles characteristic of the ring-stage parasite, where it remains in the vacuolar system until they fuse to form the single food vacuole of later stages.

Our results indicate that in early and mid-stage rings, endocytic vesicles function as individual food vacuoles, each able to break down hemoglobin and generate hemozoin crystals (see [29], [57]), and that in late rings there is a switch to a single vacuole, indicating that new molecules able to facilitate vacuolar fusion are functional at this developmental threshold. Our IFA and EM/IEM results clearly indicate that this switch is achieved by the fusion of small food vacuoles rather than the formation of a completely new large single vacuole as proposed by Elliot et al. [57]. This is demonstrated by the IFA and IEM labeling of progressively larger but fewer food vacuoles in rings, and the strong MSP1_19_ labeling of all vacuoles containing hemozoin in rings and later stages - these findings would be unexpected if there is a major, separate endocytic event responsible for forming the single food vacuole at the end of the ring stage.

Our IFA and IEM results therefore confirm and extend the findings of Drew and colleagues [15] that, in late rings, MSP1_19_ is associated with the food vacuole. However, the IFA evidence for colocalized foci of MSP1_19_ and MSP8 at the parasite's surface in ring stages reported by these authors needs to be re-interpreted in view of our IEM findings. At this much higher level of resolution, it is clear that these apparently surface concentrations correspond to small peripheral food vacuoles within the parasite.

The IEM results show that after endocytosis, MSP1_19_ becomes closely located with the lining membrane of the small food vacuoles and retains this position in the single vacuole of later stages, indicating that this molecule, and it is likely, the portion of surface membrane to which it is GPI-anchored, become permanently incorporated into the vacuole wall membrane. There is evidence that GPI-anchored merozoite coat proteins such as MSP1 are associated with cholesterol-rich lipid microdomains (rafts) [58], which therefore could be endocytosed into the vacuolar system from the ring surface with MSP1_19_. The persistence of this fragment may therefore also reflect an equal persistence of the associated lipid raft components in the food vacuole wall.

The IEM results additionally show that in the single vacuole of trophozoites and early schizonts, the molecules of MSP1_19_ are clustered into one or more patches of high concentration on the vacuole wall, rather than being spread evenly around its perimeter. This suggests that MSP1_19_ molecules and the membrane lipids with which they associate form a coherent unit integrated into the vacuole wall membrane, preserving a group identity separate from other membrane components of the wall. This behavior implies some form of lateral interaction between MSP1_19_ molecules either directly with each other or indirectly through clustering of their linked membrane lipids. Interestingly, inspection of published IEM reports of CRT localization to the food vacuole wall [30], [59] also show a patchy distribution. Additionally, double IFA labeling for MSP1_19_ and CRT does not show a total colocalization, suggesting that they cover overlapping or different areas of the food vacuole wall.

These findings indicate that the membrane lining the food vacuole may be a mosaic of different molecular compositions, of which MSP1_19_-attached membrane is only a part. Such an arrangement may not be too surprising, considering what must be the highly dynamic nature of this boundary, which constantly receives new membrane from the cytostomes and probably by fusion of Golgi-derived vesicles carrying proteolytic enzymes [25], yet at the same time maintaining close control over its ionic contents by active transport across its wall.

It may also be significant that during schizogony the vacuole shrinks in diameter. At first it appears that the lining membrane becomes folded during this process, but later it loses its folds and it is likely that its surface area is reduced by some other mechanism. It is significant that as the food vacuole becomes smaller, the proportion of the wall labeling for MSP1_19_ increases until at the end it covers most or all of the inner surface of the food vacuole (now the residual body) (see Figure 8C–F). As shown by Jackson et al [52], during the schizont period the internal membranes of the food vacuole vanish and at the same time the lipid body appears on the vacuole's periphery, suggesting mobilization and retrieval of lipid from the food vacuole interior. A similar process could also account for the loss of membrane from the vacuole wall, although in this case it would be selective, leaving membrane associated with MSP1_19_, as its chief component.

Besides the potential functional importance of MSP1_19_ the results reported here throw new light on the process of endocytosis into the food vacuole at different stages of the cycle. It is plain that food vacuoles formed in rings are in a number of respects different from those of later stages. The avid uptake of MSP1_19_ from the ring surface contrasts strongly with the absence of uptake of full length MSP1 into the schizont food vacuole, as shown by the failure of N-terminal-directed antibodies to detect vacuolar MSP1. It is likely that both GPI-anchored forms of this molecule are associated with cholesterol-rich lipid microdomains [58] and that in rings, MSP1_19_ is carried into the vacuole by association with such specific lipids. In later stages when it would be wasteful for the parasite to allow vacuolar degradation of newly synthesized GPI-anchored surface proteins destined for the next brood of merozoites, some mechanism must exist at the mature cytostome for ensuring MSP1 is excluded from endocytosis. The molecular basis for this selective process is not at present apparent.

It is possible that the presence of MSP1_19_ in food vacuoles is merely an aspect of the food vacuole's ability to store undegradable biomolecules, already evident in hemozoin sequestration. However, as MSP1_19_ is located at the inner surface of these organelles, it is likely that any function relates to that boundary. There is experimental evidence that some anti-MSP1_19_ antibodies (though not the anti-MSP1_19_ mAb 1E1 used here) carried into the red cell on the surface of the merozoite can interfere with intracellular development [60]–[62]. The basis for this interference is unknown but warrants further investigation. Moreover, the location of MSP1_19_ along the food vacuole wall rather than in isolated clumps or vesicles within its lumen (as typical of degraded structures in secondary lysosomes of many eukaryotes: (see [63] for a review)) argues that MSP1_19_ is not merely discarded as a functionless end-product.

The protease-resistant structure of MSP1_19_ suggests that it may serve as a protective protease-resistant protein coat on the inside of the food vacuole, guarding other membrane proteins against the powerful proteases within this organelle. As it covers only part of the wall, it is unlikely to be the only protective agent, and other molecules with similar resistance may also participate, including MSP8, which has a similar EGF domain structure. However, gene disruption experiments have shown that MSP8 is not essential for parasite progress [15], [64] so additional protease-resistant proteins may be necessary to provide adequate protection.

Another possibility is that MSP1_19_ might be involved in nucleating hemozoin crystallization, a role posited for a number of parasite molecules including HRPII, which is either transported directly to the food vacuole or first trafficked to the red cell cytoplasm and then taken back into the food vacuole during feeding [65], [66]. Current evidence indicates that neutral lipid droplets within the food vacuole are important for hemozoin formation [67] although other molecules might participate. It would be useful to know if MSP1_19_ has the capacity to nucleate or accelerate hemozoin crystallization.

A third possibility is that by virtue of its association with membrane lipids including those forming cholesterol-rich lipid microdomains, MSP1_19_ is important in mechanically stabilizing the vacuole membrane especially in late schizonts when the vacuole is packed with sharp-ended hemozoin crystals. Further experiments are needed to test these possibilities.

Whatever the true function, the findings reported here point to an important but as yet undefined role for merozoite proteins in the intracellular phase of the parasite's erythrocytic cycle, and they also emphasize our lack of understanding of the degradative pathways within the parasite. Both of these aspects of the parasite's biology deserve attention as potential targets for the development of antimalarial strategies.
